# Reply to Dr. Peters

**Published:** 1892-01

**Authors:** D. E. Salmon

**Affiliations:** Chief of the Bureau


### REPLY TO DR. AUSTIN PETERS' CRITICISM OF THE BUREAU OF ANIMAL INDUSTRY.

By Dr. D. E. Salmon, Chief of the Bureau.

At the meeting of the United States Veterinary Medical Association, held in Washington on September 15 and 16, 1891, a meeting from which the writer was unavoidably absent, Dr. Peters, as chairman of the committee on Intelligence and Education, presented a report in which he bitterly assailed and criticised the Bureau of Animal Industry, and especially the writer, as the chief of that Bureau. This report has since been published, and has, no doubt, been perused, in whole or in part, by the readers of The Journal; at any rate, it has been spread before the world as being the deliberate statements of one of the most important committees of our National veterinary organization. The assertions contained in it are of such a nature that they cannot be allowed to pass unnoticed either by the individual whose name and reputation are involved, or by the association before which the charges were so publicly made.

If Dr. Peters' report is true, then the writer of this communication is an unworthy member of the Association ; if it is false, then the chairman of the committee on Intelligence and Education is guilty of unprovoked, deliberate and malicious slander. In either case the self-respecting members of the Association cannot allow the matter to stand without taking some positive action. To act intelligently they must have both sides of the controversy before them, and I have consequently prepared this reply to Dr. Peters' statements, which I shall send for publication to the two veterinary periodicals of the country.

All will agree with me, no doubt, that a person who is assailed in such language as appears in Dr. Peters' contribution should, in common fairness, be accorded an opportunity to make a reply before he is condemned, even if there were nothing at stake but his individual reputation. And when, as in this case, there is in addition to individual honor the reputation of a great Bureau of the National Government, a Bureau on the force of which the veterinary profession has always been largely represented, how much greater reason is there for expecting the fullest hearing, and the most unbiased treatment.

Dr. Peters, in his paper, endeavored to give the impression that he boldly made these charges in my presence, since he says that he is glad that he has deferred the matter until this year, "as it has given me (him) an opportunity to beard the lion in his den, so to speak, which I (he) always prefer to do, if the opportunity permit." Instead of this being the case, he seized the opportunity when he knew I was unavoidably absent in Europe, representing the Secretary of Agriculture at the International Congress of Hygiene and Demography at London, and at the International Agricultural Congress at The Hague, to make an attack not only on the scientific work of the Bureau, but upon my honesty and truthfulness. This attack was the more cowardly since he also knew that Dr. Smith, who has charge of the investigations, was not a member of the Association, and was therefore not in a position to reply.

It should be remarked in this connection that Dr. Peters has adopted three lines of attack. The first and chief one consists of personalities; the second is the misrepresentation of our views in order to secure plausible subjects for criticism; while the third and least prominent is the attempt to controvert views which we have actually expressed. It is fortunate that this whole subject is a matter of record, and that all the evidence has been printed where it is easily accessible. There is, consequently, no excuse for misrepresentation or inexact statements.

The personalities are chiefly based on the assumption that I have failed to give Dr. Smith credit for the part which he has had in the investigation of swine diseases. "In a special report of the Bureau of Animal Industry upon 'Hog Cholera: Its History, Nature and Treatment,' issued in 1889," Dr. Peters says, "there is a short history of the investigations of swine disease made in the United States, but we do not find any mention of the name of Billings, although he discovered at once the bacterium which the chief of the Bureau of Animal Industry had been searching for, for years, and which he probably would not have found for some time if he had not had the help of an assistant whom he was not generous enough to credit with the discovery, and so let it pass as his own."

We will now go to the records and see how much credit has been given to Dr. Smith for the work which he has done. In the report of the Bureau for 1885, page seven, is the following sentence: "I have been ably assisted in these investigations by Dr. Theobald Smith, whose untiring services have been indispensable and invaluable; also, by Dr. F. L. Kilborne, who has had charge of the experiment station." In 1886, long before the report for that year was issued, and in order to remove any possible doubt as to the manner in which the investigations were conducted, I published an article in which the following paragraphs occur :

"Very soon after these experiments" (those of 1884) "were made, the many duties which devolve upon me as chief of the Bureau of Animal Industry made it necessary to place the investigations of hog cholera almost entirely in the hands of my assistant, Dr. Theobold Smith, in whose ability and devotion to the work I had and still have the utmost confidence. While, therefore, I marked out the lines of investigation, and kept a personal supervision over what was done, the work has been carried out by another.

"Now one of the very first results of this arrangement was the conclusion by Dr. Smith that hog cholera was caused by a motile bacterium, which certainly was a different germ from the one that I had described in 1884." (Breeders' Gazette, Nov. 11, 1886.)

The very article in which these sentences occur was referred to by Dr. Frosch, and this reference was reproduced by Dr. Peters, so there can be no excuse for either of them to plead ignorance of its contents; and yet, in spite of this plain statement, Dr. Frosch, after mentioning the article, pretends that he did not know until the publication of Dr. Smith's recent paper, the part which the latter (Smith) had taken in the work. And Dr. Peters, taking his cue from Frosch, chimes in with the same order of statements.

This is by no means all the credit that has been given. In the report of the Bureau for 1886, page 7, I said : "For the careful and accurate manner in which the experiments referred to above have been carried out, I am indebted to Dr. Theobald Smith, director of the laboratory, and to Dr. F. L. Kilborne, director of the experiment station, both of whom have shown the most commendable activity and interest in the work, and whose intelligence and zeal have enabled us to satisfactorily decide some of the most difficult questions which modern science has been called upon to elucidate." It will be noticed that although Dr. Smith came to me a young man, I had very soon made him director of the laboratory, and I recognized in my reports not only his work, but the position which he filled. This report for 1886 was the one which contained the first record of his work in connection with the disease which we call swine plague ; and we therefore have the most explicit evidence that he received full credit for his work with both diseases.

In the Bureau report for 1887 and 1888, which was published in one volume, I said (page 7): "In conclusion, it affords me pleasure to acknowledge the ability, energy and devotion to work shown by Dr. Theobald Smith and Dr. F. L. Kilborne in conducting the experimental work, the details of which are given in this report." In the Special Report on Hog Cholera, mentioned by Dr. Peters in the quotation made above from his paper, occurs the following paragraph:

"The greater part of the detailed study of the disease, the planning of the experiments, and the bacteriological investigations have been carried out by Dr. Theobald Smith, while the conducting of the experiments, the care of the experimental animals and the general management of the experiment station have been under the direction of Dr. F. L. Kilborne. I can only speak in the highest terms of the untiring industry and skill displayed by both of these gentlemen."

It is unnecessary for me to make further quotations to establish the fact that Dr. Peters was guilty of the most inexcusable misrepresentation and distortion of facts in what he said in reference to this matter. "You will see," he says, "that Jeffries in his paper gives Smith the credit for the work he has done." But why should he not have given him credit? Were not all the facts detailed with sufficient minuteness in the reports, as shown by the above quotations? Again, he says: "It has been no secret to me for the last year and a half as to who was actually conducting these investigations in the Bureau of Animal Industry." Why should it have been any secret to him or to any one else who consulted the reports of the Bureau as to the exact position and work of each one connected with the investigations?

There is one other paragraph in Dr. Peter's paper which I must quote and comment on in this connection. He says: "If the Bureau of Animal Industry is to be a political organization, why not have its chief simply write the letter of transmissal of his annual report to the Secretary of Agriculture, and have a few true scientists in his employ to work unhampered, and make their own reports upon the questions that they have been studying upon. This, at least, for the sake of making a more creditable appearance to other civilized nations, if we have no respect for ourselves."

This is very strong language, calculated and intended to discredit the bureau and those connected with it both at home and abroad. The reader will agree with me, I am sure, that it should not have been published broadcast without the most ample evidence that the imputation which it conveys was fully justified by the facts. No one can have any excuse for being ignorant of the fact that the Bureau of Animal Industry is not a political organization in the sense in which that term is here used. The law provides that the chief "shall be a competent veterinary surgeon," thus taking the appointment as far as possible out of the realm of politics. The chief has no power to make appointments or dismissals, and, consequently, there is no political work for him to do, and no excuse for his being a politician. The present chief was appointed by the late Dr. Loring, in 1884, without any political influence whatever being brought to bear, and solely because of his competency as a veterinary surgeon, and his qualifications to perform the arduous and responsible duties of the position. He has served under three administrations, two of which were Republican and one Democratic, and there has been no effort of any head of the Department to displace him, or any of his scientific staff. How different this would have been if the Bureau had been in any sense a political organization.

More than this, I adopted the plan of transmitting the reports of Dr. Smith and the other scientists under my direction as soon as it appeared proper to do so. By consulting the report of the Secretary of Agriculture for 1889, page 75, it will be seen that the report of these investigations is introduced as follows: "The following brief account of the investigations, conducted under my direction, into the nature of infectious animal disease has been prepared by Dr. Theobald Smith, who is in charge of this branch of the work of the Bureau of Animal Industry." In the report of the Secretary of Agriculture for 1890, page 105, I have introduced Dr. Smith's report in precisely the same language as that just quoted. After this plan had been adopted by me and carried out for two years, Dr. Peters appears upon the scene and trys to convey the impression that he is the first to suggest it; that it has never been adopted, and that we are deserving of the contempt of other nations and our own people, because it has not been done.

There are other personalities in Dr. Peters' paper which are equally unworthy of him and of the committee for which he spoke. They might be taken up one by one and shown to be just as uncalled for, just as far from the truth as those which have been considered. I will not weary your readers, however, by going further into this subject, since his whole case hangs upon his assumption that I failed to give Dr. Smith credit for his work in the investigation of swine diseases. This assumption of Dr. Peters is conclusively disproved by the quotations which I have made from my reports, and shown to be without reason or justification ; and that being the case, the inferences and insinuations which he bases upon it must necessarily be taken as equally unworthy of belief.

Having disposed of the first line of attack, the personalities, let us briefly consider the second line, that is, the misrepresentations of our views on scientific questions, made for the purpose of introducing plausible arguments for strengthening the weak parts of his paper. It is simply the well-known dodge of polemical writers, generally referred to as setting up straw men for the moral effect which follows from knocking them over.

Dr. Peters quotes from the report of the Bureau for 1886, the passage which explains that we had differentiated two diseases of swine that had previously been regarded as one, and that it was consequently necessary to prevent confusion in the future to apply a distinctive name to each, and that, after full consideration, it had been concluded best to call the disease described in the report of 1885, "Hog Cholera," and that described in the report of 1886 "Swine Plague," and that this was the more desirable, since the latter disease existed in Germany, where it was also called swine plague (schweine-seuche.) These reasons certainly appear to be sufficient for our course, and the man who sees a hidden intent on our part to cause confusion, instead of preventing it, must have an extremely suspicious nature. And yet the doctor seriously states that the following questions propound themselves to him after reading our explanation :

"After speaking of the disease as swine plague for several years" (one year to be exact.—D. E. S.) "did the chief of the Bureau of Animal Industry call Billings' swine plague 'hog cholera' for the sake of creating confusion." If Dr. Peters will refresh his memory as to the literature of the subject, he will find that Dr. Billings' report on Swine Plague did not appear until June 30, 1888, or a year and a half after the report of the Bureau that he quotes from was written, and that we could not have known in advance what his position would be, or what name he would apply to either disease. In his newspaper articles Dr. Billings asserted most positively that the germ he had found was identical with that described as causing the German swine plague (schweine-seuche), and he repeats this in his report (1888), and goes so far as to say (page 136) that "Mr. Salmon's specific 'hog cholera microbe' was missed, and 'it ever will be missed' in the American swine plague, no matter who seeks it, or how much time they may spend in the hunt."

If Billings had been right in this, and his subsequent course had been consistent, there would have been no confusion, for the German swine plague, the swine plague of the Bureau and Billings' swine plague would have been caused by identical germs, and there would have been no confusion in classing the three together as one disease, or as variations of one disease. As far as could be seen, therefore, from the literature at hand at the time our report was written, our nomenclature was calculated to avoid any confusion, even with Billings' writings. We could scarcely be expected to foresee that Billings would discard his swine plague germ, which he asserted was identical in its "micro-morpho-biological phases" (Report, pages 113, 114) with the germs of "Wild Seuche," hen cholera and rabbit septicæmia, and come to the front with an entirely different microbe as the cause of his swine plague. This other microbe is the hog cholera microbe of the Bureau, as Peters sufficiently shows, and it is the germ which Billings, up to 1889, asserted had no existence. If, therefore, confusion has been the result, the Bureau of Animal Industry is not responsible for it.

"If the name 'hog cholera' was not used in the place of swine plague for the purpose of creating confusion," says Dr. Peters, "why was a septic pneumonia of the pig termed 'swine plague,' unless for the purpose of causing further confusion? When, as we have seen, the disease is not confined to swine, but a little careless (careful?) study would have shown that pigs could easily communicate it in other species of animals." As we have already explained, we called the American disease swine plague partly because an apparently identical disease in Germany, caused by an apparently identical germ, was known the world over as swine plague. That disease in Germany was described by competent men, and was accepted by practically the whole scientific world as properly described and named. If our disease is a septic pneumonia, the German swine plague is a septic pneumonia; if our disease is communicable to other species of animals, the same has been recognized as true of the German swine plague. And yet no scientist has attacked Loeffler or Schutz as being guilty of any impropriety in calling their disease swine plague. It has been reserved for Dr. Peters to make the discovery that it is a gross error to name a septic disease of pigs, communicable to other animals, swine plague; but why does he confine his remarks to the Bureau of Animal Industry, when we were only following the example of the distinguished European scientists to whom reference has just been made?

Dr. Peters says: "I think that Jeffries' work is particularly accurate, and very valuable, and am surprised that it has not attracted a great deal of attention, although it does not appear to have done so." I am ready to agree that Jeffries' paper was a valuable and timely contribution to the literature of swine diseases, but how does it happen that Dr. Peters, while so mercilessly criticising the work of the Bureau and praising the work of Dr. Billings, fails to point out that Jeffries absolutely disproves Billings' assertions that (1) the germ of the American hog cholera is identical with that of the German swine plague; that (2) the germ of hog cholera described by us in 1885 does not exist; that (3) there is only one communicable disease of swine in the United States, and that (4) a disease identical with the German swine plague does not exist here. In his position on each of these important questions, Jeffries fully confirms the work of this Bureau, yet Peters has not a word to say to that effect!

To show the superiority of the work of investigators outside of the Bureau, Dr. Peters says: "Dr. Billings boldly announces that he found his germ of swine plague in July, 1886, among the first pigs that he examined in Nebraska which had died of the disease." It should be remembered in this connection that Dr. Billings had the advantage of our report, in which we had described the germ of hog cholera, how to obtain and cultivate it, and its effects when inoculated in the smaller animals. All he had to do was to follow the methods we described in order to obtain the germ from the first hog he examined which was affected with the disease. But Billings has boldly announced a great many discoveries that have not fulfilled the expectations of himself or his friends. If he really discovered in July, 1886, the germ which he now produces as the cause of his swine plague, how does it happen that for two years afterwards he claimed that this germ had no existence? How does it happen that he asserted so positively the morphological and biological identity of the germ he then had with the *schweine-seuche* germ of Germany? How does it happen that the first germs he sent abroad were not the same as our hog Cholera germ or his present swine plague germ (Dr. E. Bunzl-Federn, Archiv f. Hygiene, XII., 1891, p. 198)? If these questions were satisfactorily answered we would be more disposed to admit the plausibility of Dr. Billings' bold announcement.

With the personalities and misrepresentations out of the way, the only statements I find remaining in Dr. Peters' paper in the nature of a serious criticism are contained in the following sentences: "The so-called swine plague of the Bureau of Animal Industry is one of those septic diseases due to filth, and is seen chiefly where putrefying city swill is fed ; and farmers around Boston find that if the swill is boiled and then fed, before there is time for putrefactive process to commence again, they are not troubled with it. In this respect it resembles closely the German *schweine-seuche.* If this be a true swine plague, make the most of it "

The objection to Dr. Peters' view as stated here is that it is too sweeping and dogmatic. His experience has been limited to a few outbreaks among hogs in the vicinity of Boston, which were chiefly fed on swill. From this limited experience with hogs kept under one set of conditions, he wishes to generalize his conclusions and make them apply to hogs in all parts of the United States and fed upon all sorts of food. And he is so intolerant toward the investigators of the Bureau that because their observations do not agree entirely with the conclusions which he has reached, he brands them as dishonest and unworthy of credence. In doing this he departs from the methods and traditions of true scientists and injures his own reputation more than he does the reputations of those whom he attacks. Whether the disease which we have called swine plague is a true plague or not depends upon the extent of territory over which it occurs, and the amount of losses from it. This disease has so far been found in Massachusetts, New Jersey, Maryland, District of Columbia, Kentucky, Illinois, and Iowa, as well as in Germany, so there is a certainty of its being widely distributed. The exact losses from it are, of course, unknown, but we have been satisfied from our investigations that they are sufficiently large to fully justify the name applied to it. Much more evidence has been accumulated to show its destructiveness here than exists in regard to it in Germany, and as has already been stated, the propriety of the name has not been questioned there.

A public official should not be unduly sensitive to criticism, nor object to it when it is reasonable and fair. But Dr. Peters has gone beyond this, attacking the honor and credibility of every scientist connected with the Bureau ; his paper, going to those ignorant of the facts, is calculated to bring reproach upon our Department of Agriculture, our scientists, our institutions, and our country. The natural consequence of such documents is to retard the progress of science, to make farmers suspicious that the funds appropriated for investigations in their behalf have not been judiciously expended, and to make it more difficult to obtain appropriations for such purposes. The Bureau of Animal Industry has employed more veterinarians, and has done more for the veterinary profession than any other institution or department of the government, and yet this man, representing for the time the profession, seized his opportunity to do what he could to criple this Bureau. And when we examine his case, as we have just done, in the light of recorded evidence, we find his argument can be characterized by the single word—misrepresentation. Condemning personalities, political methods and lack of honesty in others, he, in the same paper, makes himself liable to all of these charges.

In concluding, the writer calls upon the members of the United States Veterinary Medical Association to give this matter special consideration, to compare the quotations which he has made with the original documents, and to decide whether their body is to be made the medium for circulating such statements as are contained in this committee's report, or whether it will insist on carrying out the objects for which it was established, viz.: the promotion of harmony and good-fellowship, the advancement of the profession, and the encouragement of honest and conscientious scientific research.

